# Roles of estrogen and its receptors in polycystic ovary syndrome

**DOI:** 10.3389/fcell.2024.1395331

**Published:** 2024-06-19

**Authors:** Yao Xu, Ziyi Zhang, Rongxiang Wang, Songguo Xue, Qian Ying, Liping Jin

**Affiliations:** ^1^ Shanghai Key Laboratory of Maternal Fetal Medicine, Shanghai Institute of Maternal-Fetal Medicine and Gynecologic Oncology, Clinical and Translational Research Center, Department of Assisted Reproduction, Shanghai First Maternity and Infant Hospital, School of Medicine, Tongji University, Shanghai, China; ^2^ Shanghai Key Laboratory of Female Reproductive Endocrine Related Diseases, Hospital of Obstetrics and Gynecology, Shanghai Medical School, Fudan University, Shanghai, China; ^3^ Reproductive Medicine Center, Shanghai East Hospital, School of Medicine, Tongji University, Shanghai, China

**Keywords:** estrogen, estrogen receptor, ovary, polycystic ovary syndrome, estrogen signaling

## Abstract

Polycystic ovary syndrome (PCOS) is an endocrine disorder characterized by abnormal steroid hormone levels in peripheral blood and poor-quality oocytes. In the ovary, androgen is produced by theca cells, and estrogen is produced by granulosa cells. Androgen is converted to estrogen in granulosa cells, with cytochrome P450 aromatase as the limiting enzyme during this process. Estrogen receptors (ER) include ER alpha, ER beta, and membrane receptor GPR30. Studies have demonstrated that the abnormal functions of estrogen and its receptors and estradiol synthesis-related enzymes are closely related to PCOS. In recent years, some estrogen-related drugs have made significant progress in clinical application for subfertility with PCOS, such as letrozole and clomiphene. This article will elaborate on the recent advances in PCOS caused by abnormal expression of estrogen and its receptors and the application of related targeted small molecule drugs in clinical research and treatment.

## 1 Introduction

Polycystic ovary syndrome (PCOS) is a common endocrine-metabolic disorder characterized by hyperandrogenism, ovulatory dysfunction, and polycystic ovarian morphology (PCOM), affecting about 9%–18% of reproductive-aged women (Mykhalchenko et al., 2017). Women diagnosed with PCOS often exhibit endocrinal abnormalities, including hirsutism, insulin resistance, and obesity (Rotterdam Eshre/asrm-Sponsored PCOS consensus workshop group, 2004; Shrivastava and Conigliaro, 2023), followed by increased risk for type 2 diabetes (DM2), cardiovascular disease, and cancers of reproductive organs (Lizneva et al., 2016). The most common clinical marker of hyperandrogenism is hirsutism (Escobar-Morreale et al., 2012), which is measured by the extent of terminal hair growth in male-like areas according to the modified Ferriman–Gallwey score (Yildiz et al., 2010; Escobar-Morreale et al., 2012). What’s more, overweight and obesity play a critical role in the development of PCOS, and weight management can markedly relieve the symptoms of hyperandrogenism, metabolic dysfunction, and ovulatory dysfunction, even with only 5% body weight loss (Kiddy et al., 1992; Holte et al., 1995; Barber et al., 2019). In addition, familial clustering studies provided strong evidence for genetic contribution to the etiology of PCOS. There is a 30%–50% risk of developing PCOS in women who have a first-degree relative diagnosed with PCOS (Kahsar-Miller and Azziz, 1998; Legro et al., 1998; Kahsar-Miller et al., 2001; Moore and Campbell, 2017). Many studies identified a set of susceptibility loci and variants in genes involved in androgen biosynthesis (*CYP11A1*, *CYP17A1*, *CYP19*, *HSD17B5,* and *HSD17B6*), androgen activity (*AR*, *SHBG*, *SRD5A1,* and *SRD5A2*), insulin signaling (*INSR*, *IRS1,* and *IRS2*), folliculogenesis (*FSHR*, *LHCGR,* and *AMHR2*) and estrogen receptors (*ESR1* and *ESR2*) (Ibanez et al., 2003; Echiburu et al., 2008; Pusalkar et al., 2009; Unsal et al., 2009; Gu et al., 2010; Chen et al., 2011; Dolfin et al., 2011; Ramos cirilo et al., 2012; Shi et al., 2012; Kosova and Urbanek, 2013; Cui et al., 2015; Hayes et al., 2015; Silva et al., 2015; Azziz et al., 2016; Zhao et al., 2016; Moore and Campbell, 2017). Although enormous progress has been made in identifying PCOS loci and candidate genes, the role and function of these genes in PCOS remain unclear.

Steroidogenesis is a fundamental human hormone metabolism process involving cytochrome P450 and hydroxysteroid dehydrogenase enzymes to produce androgens and estrogens (Bondesson et al., 2015). Endocrinal disorder, especially high serum androgens, is thought to be the critical factor of PCOS. In the ovary, granulosa cells convert androgens to estrogens, and estrogen functions are mainly mediated through estrogen receptors (ERs). Three estrogen receptors have been identified in different tissues and intercellular locations: estrogen receptor alpha (ERα), estrogen receptor beta (ERβ), and the G-protein coupled estrogen receptor (GPER) (Gibson and Saunders, 2012; Yu et al., 2022). Studies in human and rodent animals have demonstrated disruption in estrogen signaling is highly related to PCOS, which makes estrogen and ERs a potential target in clinical treatment (Ryu et al., 2019). In non-human primates, it is reported that naturally hyperandrogenic female rhesus monkeys exhibit traits typical of women with PCOS (Abbott et al., 2017). Thus, anti-estrogen clomiphene citrate and aromatase inhibitor letrozole have become the first-line drug therapy to induce ovulation in PCOS treatment, and both drugs achieved good pregnancy outcomes (Legro et al., 2014; Roque et al., 2015). Here, we briefly review the roles of estrogen and its receptors in the pathogenesis and treatment of PCOS.

## 2 Polycystic ovary syndromes: definition and diagnosis

PCOS is a highly prevalent and heterogeneous disorder in women. The prevalence of PCOS ranges from 9% to 18% in reproductive-aged women due to different diagnostic criteria (March et al., 2010; Yildiz et al., 2012; Bozdag et al., 2016), which makes it the most common endocrine and metabolic disorder in women (Conway et al., 2014; Escobar-morreale, 2018). PCOS was first described by Stein and Leventhal in 1935, with the clinical characterization of oligo-amenorrhea, hirsutism, acne, obesity, and polycystic ovaries (Stein and Leventhai, 1935; Azziz and Adashi, 2016; Rosenfield and Ehrmann, 2016). Furthermore, patients with PCOS also demonstrate disorders, including insulin resistance (IR), increased risks for type II diabetes (DM2), and cardiovascular disease (Norman et al., 2001; Deugarte et al., 2005; Krentz et al., 2007; Lizneva et al., 2016; Chen and Pang, 2021). Thus, PCOS has become a prominent health issue of reproductive women, which seriously influences their physical and moral integrity. Three sets of diagnostic criteria for PCOS have been developed over the past three decades (Table 1) (Zawadzki et al., 1992; Rotterdam, 2004; Azziz et al., 2006a; Teede et al., 2023).

**TABLE 1:** Diagnostic criteria for PCOS.

	NIH 1990	Rotterdam 2003	AE-PCOS society 2006	International evidence-based guideline 2023
Criteria	1. hyperandrogenism 2. anovulation	1. hyperandrogenism 2. oligo or anovulation 3. polycystic ovarian morphology	1. hyperandrogenism 2. oligo or anovulation 3. polycystic ovarian morphology	1. hyperandrogenism 2. ovulatory dysfunction 3. polycystic ovaries on ultrasound; and in 2023, alternatively anti-Müllerian hormone (AMH) can be used instead of ultrasound
Criteria required	1 and 2	1 and 2/1 and 3/2 and 3/1 and 2 and 3	1 and 2/1 and 3/1 and 2 and 3	1 and 2/1 and 3/2 and 3/1 and 2 and 3

## 3 Estrogens and estrogen receptors

### 3.1 Steroidogenesis of estrogens

Steroidogenesis refers to a complex biochemical reaction in which steroids are generated from cholesterol under the action of hydroxysteroid dehydrogenase (HSD) and cytochrome P450 enzymes. As typical steroid hormones, sex hormones can be divided into androgen and estrogen (Moravek et al., 2015). Despite the different functions of androgen and estrogen, they are structurally similar and can be transformed from each other in the body. In addition, a certain proportion of the two sex hormones exist in balance in both men and women.

Similar to other steroid hormones, estrogen has many target tissues, which not only affect the growth, differentiation, and function of organs such as the ovary, uterus, and breast, but also play a role in the nervous system, cardiovascular system, and bone tissue (Nilsson and Gustafsson, 2011). Three major estrogens are produced through steroidogenesis, namely, estrone (E1), estradiol (E2), and estriol (E3) (Caiazza et al., 2015).

**FIGURE 1:**
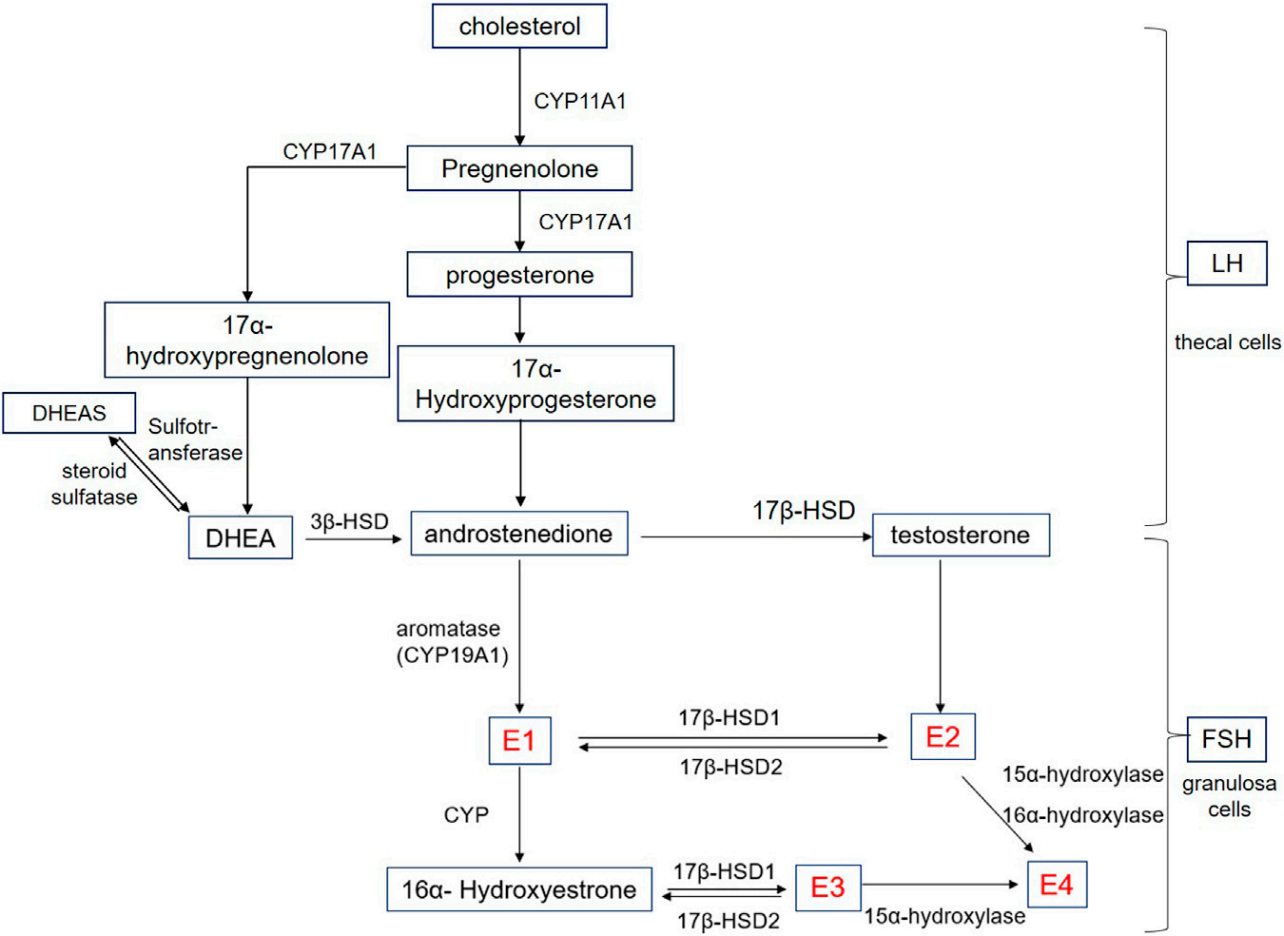
Schematic representation of estrogen biosynthesis. E1: estrone; E2:17β-estradiol; E3: Estriol; E4: Estetrol; Cyp: Cytochrome P450; HSD: Hydroxysteroid dehydrogenase; HEAS: Dehydroepiandrosterone sulfate; DHEA: Dehydroepiandrosterone; LH: Luteinizing hormone; FSH: Follicle stimulating hormone.

The biosynthesis of estrogens is shown in Figure 1, and the synthesis process is also linked to each other. In the first stage of steroidogenesis, cholesterol is converted to androstenedione under the action of CYP11A1, CYP17A1, and 3β-HSD. Among them, CYP11A1 catalyzes the cleavage of the cholesterol side chain and the conversion to pregnenolone. Furthermore, with the activity of l7, 20 carbon chain lyase, CYP17A1 catalyzes the l7α-hydroxylation of pregnenolone and progesterone. As for 3β-HSD, which is known as 3β-hydroxysteroid dehydrogenase, controls critical steroid hormone-related reactions in the adrenal cortex, gonads, placenta, liver, and other peripheral target tissues and affects all types of steroid hormones (Rasmussen et al., 2013). In the second stage, androstenedione is converted to E1 by aromatase CYP19A1, which is known as estrogen synthase and is specifically expressed in large antral and preovulatory follicles (Stocco, 2008). During the antral follicle growth, estrogen production results from follicle stimulating hormone (FSH)-dependent activation of aromatase (Dewailly et al., 2016).

On the other hand, androstenedione is converted to testosterone by 17β-HSD and is further converted to E2 by aromatase catalysis. Known as a group of enzymes, l7β-HSD are widely distributed in various tissues of the human body, including steroid hormone secretion tissues such as the placenta and ovary, and also distributed in other tissues such as fat and skin, breast, endometrium, liver, and other tissues (Vihko et al., 2001; Hilborn et al., 2017). In addition, the transformation between E1 and E2 is also catalyzed by l7β-HSD.

E3 is a steroidal estrogen produced by the human placenta and is derived from dehydroepiandrosterone (DHEA) and its sulfate (DHEAS) (Legrain et al., 2000), which are the most abundant steroid hormones in human blood circulation. Origin from the adrenal glands of the fetus and the mother, DHEAS and DHEA can be converted to each other by steroid sulfatase and sulfotransferase, respectively. In addition to the three estrogens, estetrol (E4) can only be produced by the fetal liver. Both E2 and E3 are the substrates for E4 biosynthesis. Of these, E2 requires 15α and 16α hydroxylases, while E3 requires only 15α hydroxylase (Warmerdam et al., 2008). In humans, E4 is the end product of steroid metabolism, and cannot be metabolized to E3 or E2, nor is there any other active product produced (Visser and Coelingh Bennink, 2008).

Among all the above, E2 is the main estrogen secreted by the female ovaries and the most abundant and effective endogenous estrogen in women before menopause. The first step of E2 synthesis occurs in the thecal cells where cholesterol is converted to androstenedione under the action of luteinizing hormone (LH). The second step refers to androstenedione entering into granulosa cells through the basement membrane. Following this, the third step occurs in granulosa cells where estrogen is synthesized by converting androstenedione to E2 under the action of FSH, which produces aromatase during development. Finally, the formed E2 is secreted into the follicular fluid and blood. In addition, the inactivation process occurs in the liver where E2 converts to E1 and E3, which combine with glucuronic acid and excrete from the urine. Among them, E1 has the strongest activity, while E2 and E3 are 10% and 1% of E1, respectively (Wang and Roy, 2008).

### 3.2 Estrogen receptors

Estrogen exerts physiological effects by binding to the estrogen receptor (ER), which is localized in the cell membrane, cytoplasm, or nucleus (Tang et al., 2019). Thus, two categories are divided according to the location: one is the nuclear estrogen receptor (nER), and the other is the membranous estrogen receptor (mER). The representative of the former includes estrogen receptor α (ERα) and estrogen receptor β (ERβ) (Paterni et al., 2014), which are located in the nucleus and regulate the transcription of downstream target genes specifically by binding to its ligand. In recent years, nER can also be found located in the cell membrane or cytoplasm, and is called the membranous component of the classical nuclear receptor. Except that, G protein coupled estrogen receptor (GPER), ER-X, and Gαq-ER are included in mER as well, which can rapidly regulate intracellular signal cascade reaction by binding to ligands (Heldring et al., 2007).

ERα and ERβ were first identified in 1962 (Hewitt and Korach, 2002) and 1996 (Kuiper et al., 1996), respectively. From the view of tissue distribution, ERα is mainly found in tissues that are commonly thought to have estrogen effects, such as the uterus, breast, placenta, liver, central nervous system, cardiovascular system, and bone tissue where the expression of E2 response genes are induced as well (Matsuzaki et al., 1999). However, ERβ mainly distributed in the prostate, testis, ovary, pineal gland, thyroid gland, parathyroid gland, pancreas, gallbladder, skin, urethra, lymphoid tissue, and red blood cells where ERα expression is rare or undetectable (Lau et al., 2000). What’s more, ERα and ERβ are co-distributed in the breast, epididymis, adrenal gland, hypothalamus, and tonsils (Paramanik et al., 2018).

In addition to the classical nuclear receptor for estrogen, a G-protein coupled receptor, GPR30 was identified in the ER-positive breast cancer cell line MCF-7 in 1997 (Tskitishvili et al., 2017), also known as G protein coupled estrogen receptor (GPER). The *GPER* gene is located on the 7th autosomal short arm p22.3 and is composed of four transcriptional splice variants, encoding 375 amino acids. As a seven-transmembrane signaling protein, GPER is widely distributed in a variety of tissue cells without specificity, such as neuroglia and vascular endothelium. Besides, its main functional area is not only located in the cell membrane but also in the nucleus, Golgi, or endoplasmic reticulum, which can bind E2, glucocorticoids, mineralocorticoids, and vitamin D receptors (Prossnitz and Barton, 2011; Prossnitz and Barton, 2014). In 2005, it was first demonstrated that E2 was competitively bound to GPER (Revankar et al., 2005). Subsequently, DHEA was found to have an antagonistic effect on GPER (Niro et al., 2012). In recent years, a large number of studies have shown that GPER regulates various physiological functions of cells in normal organs such as the uterus, ovary, and breast. At the same time, its abnormal expression plays a role in the occurrence and development of gynecological diseases and other tumors as well (Prossnitz and Barton, 2023).

### 3.3 ER signaling

**FIGURE 2:**
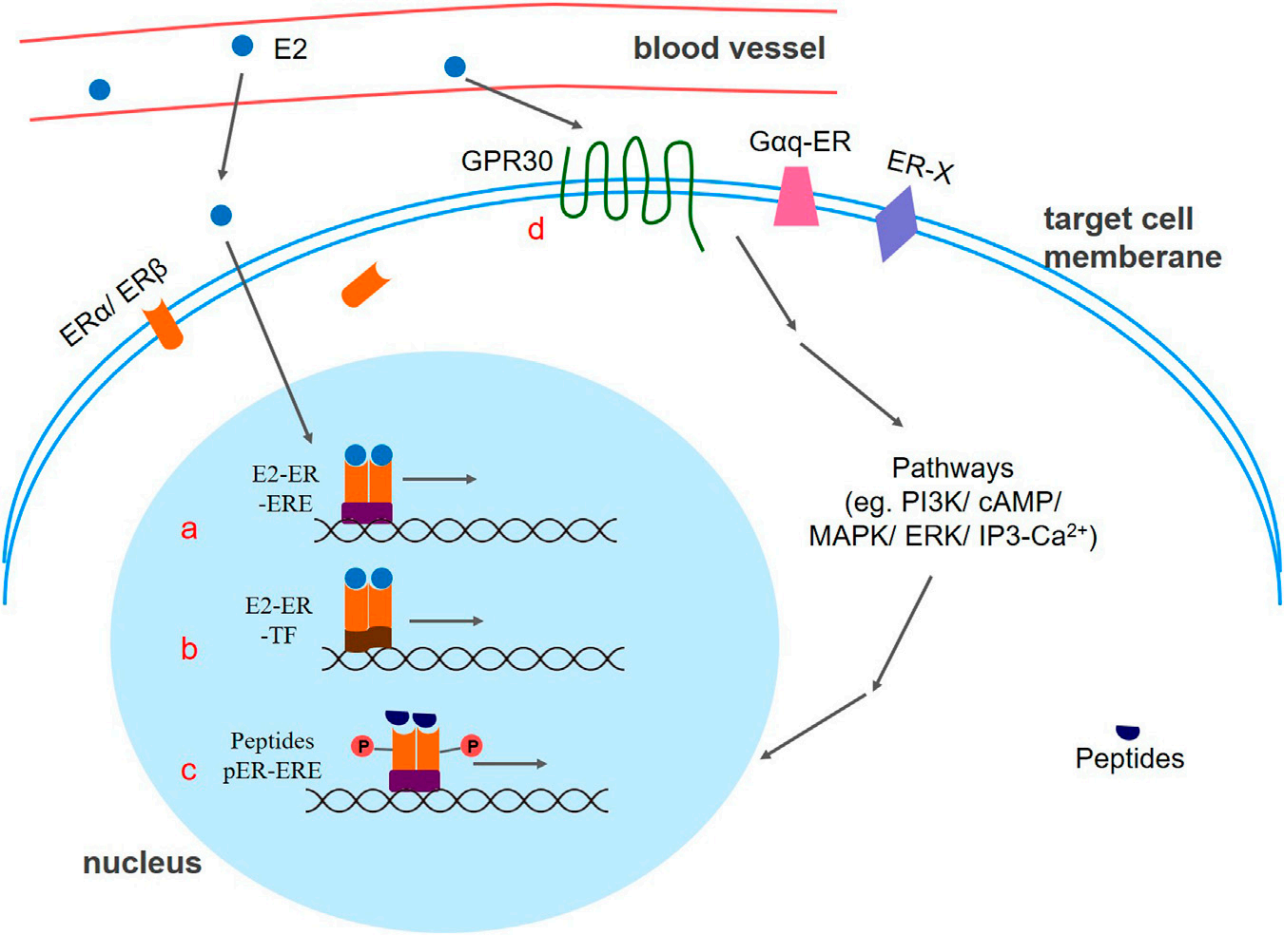
Different patterns of estrogen receptor signaling. (a) Classic ERE genomic pattern: The E2-ER complex acts as a transcriptional activator that promotes gene expression. (b) ERE independent genomic pattern. E2 activated ER binds with other classes of transcription factors, thereby regulating gene expression. (c) Ligand independent genomic pattern. Peptides such as EGF, and IGF-1 can activate estrogen receptors to express target genes. Phosphorylation of estrogen receptors may be the key factor. (d) mER-mediated signaling pathway. E2-mER mediated signal transduction causes a rapid regulation effect of the cellular response. E2:17β-estradiol; ER: estrogen receptor; GPR30: G protein coupled estrogen receptor; ERE: estrogen response element; TF: transcription factor; P: phosphate group.

There are two main types of estrogen receptor signaling pathways (Figure 2). One is Nuclear-Initiated Steroid Signaling (NISS), the genomic mode of action, which includes the classical Estrogen Response Element (ERE) genome pattern, ERE independent genomic pattern, and ligand independent genomic pattern. The other is Membrane Initiated Steroid Signaling (MISS), a non-genomic mode of action, which activates the MAPK pathway, the PI3K/AKT pathway, the cAMP/PKA pathway, and the Ca^2+^ pathway.

#### 3.3.1 Classic ERE genomic pattern

Similar to other nuclear receptor superfamily members, nER contains five functional regions: the DNA binding region (DBD), the ligand binding region (LBD), the hinge region, and two transcriptional activation regions (AF1 and AF2 regions) (Wu and Loverde, 2019). Usually, nER is in an inactive state, and exists as an oligomer complex binding with heat shock protein Hsp90 in the cytoplasm. When E2 appears and binds to the AF2 domain, nER is activated and dimerized with Hsp90 releases, thereby exposing the dimerized surface and the DBD region, which could bind the DNA reaction element ERE to the target gene. To stabilize the binding, the DBD monomer of the nERs needs to bind to two-half arms of the ERE. After ER allosteric, a hydrogen bond network is formed between the ERE and amino acid residue of the receptor to achieve selective contact of the bases. Furthermore, different cofactors are recruited to the ER-ERE complex site, thereby realizing the transcriptional regulation mechanism of different ERE genes. Thus, the estrogen-ER complex acts as a transcriptional activator that promotes gene expression (Cunningham et al., 2014; Jacquot et al., 2017). In addition to estrogen, ER ligands also include Selective Estrogen Receptor Modulators (SERMs) and Selective Estrogen Receptor Downregulators and/or Degraders (SERDs), which exerts agonism or antagonism through ERα phosphorylation.

#### 3.3.2 ERE independent genomic pattern

Even if the promoter region does not carry an ERE, estradiol can also affect the expression of related genes (Maneix et al., 2015). In this case, E2-activated ER does not bind directly to DNA but rather binds by interacting with other classes of transcription factors, thus regulating gene expression. For example, nER can bind to the AP-1 site on DNA by interacting with Fos and Jun. In addition, the ERα-Ap1 complex can also bind to the GC-rich promoter sequence. It is worth noting that ERα activates the transcription after binding to estrogen at the AP-1 site, while ERβ inhibits it. However, the antagonists tamoxifen and raloxifene become potent transcriptional agonists after binding to ERβ at the AP-1 site (Paech et al., 1997).

#### 3.3.3 Ligand independent genomic pattern

In addition to estrogen, peptides such as EGF (epidermal growth factor), IGF-1 (insulin-like growth factor-1), and 8-bromo-cAMP can also activate ERs to express target genes (Ueda et al., 2006). It is reported in the literature that a key factor in this process may be caused by phosphorylation of ERs by kinases in cells (Legrain et al., 2000). Both ERα and ERβ are phosphorylated by MAPK, but the phosphorylation sites are different. The 118th serine in the ERα AF-1 region is phosphorylated by MAPK (mitogen-activated protein kinase) after receiving EGF, resulting in the binding of the p68 RNA helicase, known as the ERα-specific coactivator, to the receptor, which finally activates the target gene (Kato, 2001). Similarly, MAPK can also phosphorylate the activation region of the ERβ N terminus, allowing the receptor to bind to the p160 coactivator SRC-1 in a ligand-independent manner and finally interact with the ERE, attenuating the transcriptional activity of ERα (Tremblay and Giguere, 2001).

#### 3.3.4 mER-mediated signaling pathway

Compared to the classical nER-mediated slow “genomic effect”, mER-mediated signal transduction is fast and cannot be inhibited by proteins and RNA synthesizers, so it is also called a fast “non-genomic effect.” That is, the binding of estrogen to ER can induce a change in the conformation of the binding site, and this conformational change can cause aggregation of some accessory proteins, thereby causing a rapid regulation effect of the cellular response.

When GPER is activated by a ligand such as estrogen or G-1, the associated G protein is first activated, and the Gαβγ heterotrimer is dissociated into Gα and Gβγ to function separately. The dissociated G protein activates or inhibits the downstream effector molecule, and changes the content and distribution of the second messenger in the cell, which acts on the corresponding target molecule through PI3K (Liu et al., 2015), Cyclic adenosine monophosphate (cAMP) (Li et al., 2010), MAPK (Filardo et al., 2002; Albanito et al., 2008), IP3-Ca^2+^ (Furuyama et al., 2014) and other pathways to exert physiological effects. For instance, Gα can activate adenylyl cyclase (AC), which induces cell signaling of second messengers such as cAMP, increases calcium ion expression, and activation of ERK signaling pathway, or enhances mitochondrial autophagy through the ERK1/2 signaling pathway. Besides, Gβγ can activate tyrosine kinase to phosphorylate, which activates matrix metalloproteinases, thereby rapidly activating ERK and other signaling pathway-associated kinases. Multiple modes of action of GPER described above have overlapping signaling pathways that mediate independent rapid intracellular signal transduction.

Apart from GPER, Gαq-ER and ER-X are newly discovered mERs, and the mechanism of signal transduction is not fully understood. Estrogen activates Gαq by binding to the receptor, then activates PLC and hydrolyzes PIP2 out of DAG to activate PKC, which subsequently activates cAMP and PKA, finally opening the potassium channel of the membrane to induce corresponding biological effects (Qiu Jronnekleiv and Kelly, 2008). Studies suggest that ER-X-mediated estrogen regulation involves the MAPK-ERK1/2 signaling pathway, and activation of ERK1 and ERK2, which is important for neuronal survival and growth.

## 4 Role of estrogen and ERs in PCOS

Estrogen and ERs exert a variety of physiological activities, including reproductive, immune, cardiovascular, endocrine, aging, and neurological diseases. The abnormal expression of estrogen and ERs is closely related to PCOS in various aspects.

### 4.1 Role of estrogen and ERs in the endometrium of PCOS

The endometrial changes in patients with PCOS vary with the level of estrogen secreted by the ovaries (Palomba Spiltonen and Giudice, 2021). When the follicles always develop immaturely, and the ovaries continue to secrete a small amount of estrogen. The endometrium of patients with PCOS is impaired progesterone response (Albaghdadi and Kan, 2021; Hamza et al., 2021). The endometrium without progesterone undergoes different degrees of hyperplasia, even the occurrence of endometrial cancer. Studies have been made to detect the expression levels of ERα and ERβ in the endometrium of patients with PCOS. Results showed that the expression of ERα mRNA and protein in the endometrium of patients with PCOS was higher than that of normal women. However the expression of ERβ was not significantly changed, suggesting that the sensitivity of PCOS intimal to estrogen stimulation is increased (Quezada et al., 2006). It is considered that PCOS intima is directly stimulated by long-term estrogen without progesterone, which may be one of the reasons for the increase of ERα expression (Gregory et al., 2002; Matthews and Gustafsson, 2003). Increased expression of ERα was observed in the endometrium of PCOS, indicating that the endometrium is more sensitive to estrogen stimulation and may affect the normal function of the mid-secretory endometrium (Giudice, 2006). However, there is little research on the expression and significance of GPER in the endometrium of infertile women, especially in PCOS patients. In summary, estrogen and some receptors play an important role in the pathogenesis of PCOS endometrium, which can be the basis for developing new drugs to alleviate the symptoms.

### 4.2 Role of estrogen and ERs in hyperandrogenemia of PCOS

Excessive androgen synthesis in the ovarian thecal cells is one of the most important features of PCOS. Hyperandrogenism participates in the pathogenesis of anovulation and may cause anovulatory infertility. And too much testosterone in serum in patients with polycystic ovary syndrome can scent into estrogen, and the ERs play a role. It has indeed been suggested that indirect, possibly ER, effects of androgens are important modulators of the reproductive characteristics of PCOS (Aflatounian et al., 2020). As mentioned above, estrogen plays an important regulatory role in the synthesis of androgen in thecal cells. It was found that the expression of *CYP17A1* in ERα-deficient follicles was significantly increased and a large amount of androstenedione was secreted after culturing wild-type follicles and ERα-deficient follicles *in vitro* (Taniguchi Fcouse et al., 2007). The results showed that ERα can reduce the production of androgen in thecal cells by inhibiting the expression of *CYP17A1*, and plays a key role in the regulation of ovarian androgen synthesis and ovarian function. Therefore, the abnormal expression of ERs in the follicles leads to changes in the estrogen sensitivity of thecal cells, which matters in the pathogenesis of ovarian hyperandrogenemia.

### 4.3 Role of estrogen and ERs in ovarian PCOS

The main pathological changes in the ovary in PCOS patients were bilateral ovarian enlargement and pearl-like cystic follicles. It was found that ERα KO adult mice display polycystic ovary, increased expression of LH receptors in ovarian follicular cells and granulosa cells, and increased concentration of serum LH, androgen, and estradiol levels (Couse et al., 2003), which indicates abnormal expression of ERα plays an important role in PCOS ovarian disease. In addition, studies showed that follicular granulosa cells in ERβ KO mice were less sensitive to gonadotropin response, and aromatase expression in granulosa cells was decreased, leading to an elevated androgen level (Couse et al., 2005). The results demonstrate that ERβ mediated estradiol actions may be the key factor responsible for maintaining differentiation of ovarian granulosa cells and the ovulation function, and lack of ERβ will destroy preovulatory follicles and finally lead to the phenotype of ovulation dysfunction. αβERKO mice ovaries demonstrate a unique morphology of the supportive-like cell, with no pre-ovulation follicles or corpus luteum formation, and granulosa cell stratification, which indicates that both subtypes of nERs are essential to maintain normal ovarian function in females. Moreover, studies have shown that GPER, along with estrogens can inhibit oocyte meiosis and mediate oocyte maturation in zebrafish (Pang and Thomas, 2010). Except, GPER has proved to be a mouse oocyte-specific estrogen membrane receptor, which can regulate oocytes to undergo meiosis again and inhibit the maturation of follicles (Li et al., 2013).

### 4.4 Role of estrogen and ERs in the metabolism of PCOS

Metabolic dysfunction is one of the most common phenotypes of PCOS, among which, insulin resistance is commonly seen in PCOS patients. The relationship between PCOS and IR was first reported in 1980, showing that obese women with PCOS have an increased insulin response compared with obese controls (Burghen et al., 1980). What’s more, Z. Douma recently discovered that the *ESR1* gene suggested correlations with a metabolic profile of PCOS by haplotype analysis. They found that four haplotypes reconstructed in the *ESR1* gene were highly associated with PCOS and one of these is consistent with insulin resistance in haplotype trend regression (Douma et al., 2020). Estrogen actions in pancreatic islet β-cells regulate insulin secretion and estrogen deficiency contributes to metabolic dysfunction prone to obesity, the metabolic syndrome, and DM2. And GPER mediates E2-stimulated pancreatic β-cell insulin (Sharma and Prossnitz, 2011; Mianowska et al., 2020). Significantly modified metabolic and endocrine features can be found in PCOS patients by using insulin-sensitizing drugs, which may demonstrate that insulin resistance plays an important role in the etiology of PCOS (Holte et al., 1995; Azziz et al., 2001; Barber et al., 2006). Meanwhile, IR, along with the subsequent hyperinsulinemia increased the risk for impaired glucose tolerance and DM2 in PCOS patients (Jayasena and Franks, 2014; Azziz, 2018). Moreover, hyperinsulinemia leads to hyperandrogenism, which further aggravates the syndrome. In addition, a study demonstrated that ERα actions confront obesity and metabolic dysfunction by mitochondrial function in adipocytes (Zhou Zmoore et al., 2020). Adipocytes express aromatase, resulting in intracrine estrogen synthesis. And GPER contributes to estrogen-dependent proliferation and lipid metabolism (Chaturantabut et al., 2019). In recent years, there have also been some findings on the increased risks of PCOS with dyslipidemia, hypertension, and other cardiovascular diseases (Azziz et al., 2006b; Cobin, 2013). Estrogen serving as a cardioprotective agent is mediated by ERα, Erβ, and GPER signaling pathways to maintain cardiovascular homeostasis (Knowlton and Lee, 2012; Aryan et al., 2020; Visniauskas et al., 2023). Thus, cardiovascular risk factors should be assessed in patients with PCOS. Perhaps estrogen and Ers are not directly related to the development of PCOS, but they are involved in the metabolic phenotype of complications of the syndrome. Therefore, genes encoding for ESRs may become valuable markers for metabolic features of PCOS at the clinical scale.

### 4.5 Role of estrogen and ERs in immune regulation of PCOS

PCOS is characterized by chronic low-level inflammation, hormonal imbalance, and immune dysregulation (Luan et al., 2022; Shamsi et al., 2022). Abnormalities in the immune system are significant directions that have recently been studied extensively for their role in PCOS (Franks and Hardy, 2006; Su et al., 2018; He et al., 2020). The function of the immune system exhibits exceptional sexual dimorphism, as shown by the active role of estrogen/ER signaling in the development, differentiation, and function of innate and adaptive immune cells (Chakraborty et al., 2023). Dendritic cells (DCs), as key antigen-presenting cells in the immune system, present antigens to different receptors on different immune cells to activate innate and adaptive immune responses (Lindquist et al., 2004; Geissmann et al., 2010). Furthermore, DCs induce a positive ovulatory response by affecting the expansion of the cumulus-oocyte complex, ovum release from the ovarian follicle, formation of a functional corpus luteum, and enhanced lymphangiogenesis (Cohen-Fredarow et al., 2014). According to a study by O. Fainaru et al., the maturity of DCs is correlated positively with ovarian function determined by the synthesis of estrogen response to gonadotropins (Fainaru et al., 2012). However, a study has shown that PCOS patients have a decreased percentage of CD11c+ HLA-DR + DCs in follicular fluid and there is a positive correlation between serum estrogen level and HLA-DR expression (Zhang et al., 2017). These studies indicate the declination of serum estrogen levels in PCOS patients may lead to dysfunction of DC maturation, thereby affecting the inflammatory response and resulting in the failure of follicle development.

## 5 Estrogen-related drugs in PCOS treatment

Routine management of PCOS includes lifestyle intervention, bariatric surgery, and drug treatment (Azziz et al., 2016; Reyes-Munoz et al., 2018; Franik Sle et al., 2022). Insulin resistance and estrogen metabolism are two typical targets of drug therapies. Metformin, a synthetically derived biguanide that was commonly used in the treatment of DM2, was used as an insulin-sensitizer drug in PCOS treatment. Metformin helps women in terms of decreasing body weight, serum insulin levels, and androgen concentrations (Naderpoor et al., 2015; Morley et al., 2017). Moreover, G-1, selective GPER agonists, increases glucose-stimulated insulin secretion in pancreatic islets of patients with DM2, while also suppressing glucagon and somatostatin secretion (Kumar et al., 2011). Combined (estrogen-progestin) oral contraceptives are the first-line medical treatment for menstrual discords and hyperandrogenism. It induces predictable withdrawal bleeding, offers reliable endometrial protection, and provides contraception (Teede et al., 2023). Clomiphene citrate (CC), an estrogen-receptor modulator, has been used for decades to induce ovulation (Misso et al., 2012). CC can increase ovarian stimulation and follicular development by blocking estradiol receptors in the hypothalamus. However, 15%–20% of PCOS patients remain anovulation after standard CC treatment, indicating CC resistance (Kurabayashi et al., 2006). Letrozole is an aromatase inhibitor and was first used to induce ovulation in 2001 in women exhibiting CC resistance (Mitwally and Casper, 2001). It blocks estrogen synthesis by inhibiting the enzyme aromatase, which can convert androgens to estrogens (Legro et al., 2014). Both CC and Letrozole are first-line drugs in PCOS treatment and there is much controversy over which therapy leads to a better clinical outcome. Legro et al. found that letrozole can achieve higher live birth and ovulation rates among PCOS women compared with CC (Legro et al., 2014). Nevertheless, Guang et al. demonstrated that the letrozole showed no advantages compared with CC in PCOS treatment (Guang et al., 2018). In a meta-analysis including seven prospective RCTs, Roque et al. found no differences in multiple pregnancy, miscarriage, and ovulation rates between Letrozole and CC groups and the letrozole group exhibited higher live birth and pregnancy rates in patients with PCOS (Roque et al., 2015). The 2023 International evidence-based guideline for the evaluation and treatment of PCOS recommends letrozole as the preferred first-line drug for infertility treatment, along with clomiphene plus metformin (Teede et al., 2023). Recently, several studies showed that melatonin, a neuroendocrine hormone secreted by the pineal gland, can mediate steroidogenesis, folliculogenesis, and oocyte maturation in the ovary (Talpur et al., 2017; Yu et al., 2019). Melatonin treatment can significantly decrease androgen and anti-mullerian hormone levels and by contrast, increase FSH levels after 6 months of melatonin treatment in PCOS patients (Tagliaferri et al., 2018). Additionally, it was found that melatonin had a positive impact on oocyte quality and *in vitro* culture of COCs from PCOS patients with melatonin can significantly increase the maturation rate of oocytes in PCOS patients (Yu et al., 2019).

## 6 Concluding remarks

PCOS is a common endocrine and reproductive disorder and metabolic dysfunction, especially hyperandrogenism is the upmost manifestation of the disease. Estrogens and ERs regulate the balance of steroidogenesis and play a significant role in the pathogenesis of PCOS. In this review, we detailed the biosynthesis of estrogens and the functions of ERs and ER signaling, trying to illuminate the essential role of estrogens and ERs in PCOS. Lastly, we reviewed estrogen-related drugs in PCOS treatment. This article exhibits a deep understanding of PCOS in the view of estrogen signaling, which may provide help in the drug screening and clinical management of PCOS.

## References

[B1] Abbott D. H. Rayome B. H. Dumesic D. A. Lewis K. C. Edwards A. K. Wallen K. (2017). Clustering of PCOS-like traits in naturally hyperandrogenic female rhesus monkeys. Hum. Reprod. 32 (4), 923–936. 10.1093/humrep/dex036 28333238 PMC6251677

[B2] Aflatounian A. Edwards M. C. Rodriguez Paris V. Bertoldo M. J. Desai R. Gilchrist R. B. (2020). Androgen signaling pathways driving reproductive and metabolic phenotypes in a PCOS mouse model. J. Endocrinol. 245 (3), 381–395. 10.1530/JOE-19-0530 32229702

[B3] Albaghdadi A. J. H. Kan F. W. K. (2021). Therapeutic potentials of low-dose tacrolimus for aberrant endometrial features in polycystic ovary syndrome. Int. J. Mol. Sci. 22 (6), 2872. 10.3390/ijms22062872 33808965 PMC7998611

[B4] Albanito L. Sisci D. Aquila S. Brunelli E. Vivacqua A. Madeo A. (2008). Epidermal growth factor induces G protein-coupled receptor 30 expression in estrogen receptor-negative breast cancer cells. Endocrinology 149 (8), 3799–3808. 10.1210/en.2008-0117 18467441 PMC2488235

[B5] Aryan L. Younessi D. Zargari M. Banerjee S. Agopian J. Rahman S. (2020). The role of estrogen receptors in cardiovascular disease. Int. J. Mol. Sci. 21 (12), 4314. 10.3390/ijms21124314 32560398 PMC7352426

[B6] Azziz R. (2018). Polycystic ovary syndrome. Obstetrics Gynecol. 132 (2), 321–336. 10.1097/AOG.0000000000002698 29995717

[B7] Azziz R. Adashi E. Y. (2016). Stein and leventhal: 80 years on. Am. J. Obstet. Gynecol. 214 (2), e1–e247. 10.1016/j.ajog.2015.12.013 26704896

[B8] Azziz R. Carmina E. Chen Z. Dunaif A. Laven J. S. E. Legro R. S. (2016). Polycystic ovary syndrome. Nat. Rev. Dis. Prim. 2, 16057. 10.1038/nrdp.2016.57 27510637

[B9] Azziz R. Carmina E. Dewailly D. Diamanti-Kandarakis E. Escobar-Morreale H. F. Futterweit W. (2006a). Positions statement: criteria for defining polycystic ovary syndrome as a predominantly hyperandrogenic syndrome: an Androgen Excess Society guideline. J. Clin. Endocrinol. metabolism 91 (11), 4237–4245. 10.1210/jc.2006-0178 16940456

[B10] Azziz R. Carmina E. Dewailly D. Diamanti-Kandarakis E. Escobar-Morreale H. F. Futterweit W. (2006b). Positions statement: criteria for defining polycystic ovary syndrome as a predominantly hyperandrogenic syndrome: an Androgen Excess Society guideline. J. Clin. Endocrinol. Metab. 91 (11), 4237–4245. 10.1210/jc.2006-0178 16940456

[B11] Azziz R. Ehrmann D. Legro R. S. Whitcomb R. W. Hanley R. Fereshetian A. G. (2001). Troglitazone improves ovulation and hirsutism in the polycystic ovary syndrome: a multicenter, double blind, placebo-controlled trial. J. Clin. Endocrinol. metabolism 86 (4), 1626–1632. 10.1210/jcem.86.4.7375 11297595

[B12] Barber T. M. Hanson P. Weickert M. O. Franks S. (2019). Obesity and polycystic ovary syndrome: implications for pathogenesis and novel management strategies. Clin. Med. insights Reprod. health 13, 1179558119874042. 10.1177/1179558119874042 31523137 PMC6734597

[B13] Barber T. M. Mccarthy M. I. Wass J. A. Franks S. (2006). Obesity and polycystic ovary syndrome. Clin. Endocrinol. 65 (2), 137–145. 10.1111/j.1365-2265.2006.02587.x 16886951

[B14] Bondesson M. Hao R. Lin C. Y. Williams C. Gustafsson J. Å. (2015). Estrogen receptor signaling during vertebrate development. Biochimica biophysica acta 1849 (2), 142–151. 10.1016/j.bbagrm.2014.06.005 PMC4269570 24954179

[B15] Bozdag G. Mumusoglu S. Zengin D. Karabulut E. Yildiz B. O. (2016). The prevalence and phenotypic features of polycystic ovary syndrome: a systematic review and meta-analysis. Hum. Reprod. 31 (12), 2841–2855. 10.1093/humrep/dew218 27664216

[B16] Burghen G. A. Givens J. R. Kitabchi A. E. (1980). Correlation of hyperandrogenism with hyperinsulinism in polycystic ovarian disease. J. Clin. Endocrinol. metabolism 50 (1), 113–116. 10.1210/jcem-50-1-113 7350174

[B17] Caiazza F. Doherty G. Winter D. C. Sheahan K. (2015). Estrogen receptors and their implications in colorectal carcinogenesis. Front. Oncol. 5, 19. 10.3389/fonc.2015.00019 25699240 PMC4313613

[B18] Chakraborty B. Byemerwa J. Krebs T. Lim F. Chang C. Y. McDonnell D. P. (2023). Estrogen receptor signaling in the immune system. Endocr. Rev. 44 (1), 117–141. 10.1210/endrev/bnac017 35709009

[B19] Chaturantabut S. Shwartz A. Evason K. J. Cox A. G. Labella K. Schepers A. G. (2019). Estrogen activation of G-protein–coupled estrogen receptor 1 regulates phosphoinositide 3-kinase and mTOR signaling to promote liver growth in zebrafish and proliferation of human hepatocytes. Gastroenterology 156 (6), 1788–1804. 10.1053/j.gastro.2019.01.010 30641053 PMC6532055

[B20] Chen Z. J. Zhao H. He L. Shi Y. Qin Y. Shi Y. (2011). Genome-wide association study identifies susceptibility loci for polycystic ovary syndrome on chromosome 2p16.3, 2p21 and 9q33.3. Nat. Genet. 43 (1), 55–59. 10.1038/ng.732 21151128

[B21] Chen W. Pang Y. (2021). Metabolic syndrome and PCOS: pathogenesis and the role of metabolites. Metabolites 11 (12), 869. 10.3390/metabo11120869 34940628 PMC8709086

[B22] Cobin R. H. (2013). Cardiovascular and metabolic risks associated with PCOS. Intern Emerg. Med. 1, S61–S64. 10.1007/s11739-013-0924-z 23494540

[B23] Cohen-Fredarow A. Tadmor A. Raz T. Meterani N. Addadi Y. Nevo N. (2014). Ovarian dendritic cells act as a double-edged pro-ovulatory and anti-inflammatory sword. Mol. Endocrinol. 28 (7), 1039–1054. 10.1210/me.2013-1400 24825398 PMC5414831

[B24] Conway G. Dewailly D. Diamanti-Kandarakis E. Escobar-Morreale H. F. Franks S. Gambineri A. (2014). The polycystic ovary syndrome: a position statement from the European Society of Endocrinology. Eur. J. Endocrinol. 171 (4), P1–P29. 10.1530/EJE-14-0253 24849517

[B25] Couse J. F. Yates M. M. Deroo B. J. Korach K. S. (2005). Estrogen receptor-beta is critical to granulosa cell differentiation and the ovulatory response to gonadotropins. Endocrinology 146 (8), 3247–3262. 10.1210/en.2005-0213 15831568

[B26] Couse J. F. Yates M. M. Walker V. R. Korach K. S. (2003). Characterization of the hypothalamic-pituitary-gonadal axis in estrogen receptor (ER) Null mice reveals hypergonadism and endocrine sex reversal in females lacking ERalpha but not ERbeta. Mol. Endocrinol. 17 (6), 1039–1053. 10.1210/me.2002-0398 12624116

[B27] Cui L. Li G. Zhong W. Bian Y. Su S. Sheng Y. (2015). Polycystic ovary syndrome susceptibility single nucleotide polymorphisms in women with a single PCOS clinical feature. Hum. Reprod. 30 (3), 732–736. 10.1093/humrep/deu361 25586784

[B28] Cunningham M. A. Wirth J. R. Naga O. Eudaly J. Gilkeson G. S. (2014). Estrogen receptor alpha binding to ERE is required for full Tlr7-and tlr9-induced inflammation. SOJ Immunol. 2 (1), 07. 10.15226/soji.2014.00107 25061615 PMC4106444

[B29] Deugarte C. M. Bartolucci A. A. Azziz R. (2005). Prevalence of insulin resistance in the polycystic ovary syndrome using the homeostasis model assessment. Fertil. Steril. 83 (5), 1454–1460. 10.1016/j.fertnstert.2004.11.070 15866584

[B30] Dewailly D. Robin G. Peigne M. Decanter C. Pigny P. Catteau-Jonard S. (2016). Interactions between androgens, FSH, anti-Müllerian hormone and estradiol during folliculogenesis in the human normal and polycystic ovary. Hum. Reprod. Update 22 (6), 709–724. 10.1093/humupd/dmw027 27566840

[B31] Dolfin E. Guani B. Lussiana C. Mari C. Restagno G. Revelli A. (2011). FSH-receptor Ala307Thr polymorphism is associated to polycystic ovary syndrome and to a higher responsiveness to exogenous FSH in Italian women. J. assisted reproduction Genet. 28 (10), 925–930. 10.1007/s10815-011-9619-4 PMC3220439 21792664

[B32] Douma Z. Dallel M. Bahia W. Ben Salem A. Hachani Ben Ali F. Almawi W. Y. (2020). Association of estrogen receptor gene variants (ESR1 and ESR2) with polycystic ovary syndrome in Tunisia. Gene 741, 144560. 10.1016/j.gene.2020.144560 32169631

[B33] Echiburu B. Perez-Bravo F. Maliqueo M. Sánchez F. Crisosto N. Sir-Petermann T. (2008). Polymorphism T--> C (-34 base pairs) of gene CYP17 promoter in women with polycystic ovary syndrome is associated with increased body weight and insulin resistance: a preliminary study. Metabolism Clin. Exp. 57 (12), 1765–1771. 10.1016/j.metabol.2008.08.002 19013303

[B34] Escobar-Morreale H. F. (2018). Polycystic ovary syndrome: definition, aetiology, diagnosis and treatment. Nat. Rev. Endocrinol. 14 (5), 270–284. 10.1038/nrendo.2018.24 29569621

[B35] Escobar-Morreale H. F. Carmina E. Dewailly D. Gambineri A. Kelestimur F. Moghetti P. (2012). Epidemiology, diagnosis and management of hirsutism: a consensus statement by the androgen excess and polycystic ovary syndrome society. Hum. Reprod. update 18 (2), 146–170. 10.1093/humupd/dmr042 22064667

[B36] Fainaru O. Hantisteanu S. Rotfarb N. Michaeli M. Hallak M. Ellenbogen A. (2012). CD11c+HLADR+ dendritic cells are present in human ovarian follicular fluid, and their maturity correlates with serum estradiol levels in response to gonadotropins. Fertil. Steril. 97 (3), 702–706. 10.1016/j.fertnstert.2011.12.030 22244783

[B37] Filardo E. J. Quinn J. A. Frackelton A. R. Bland K. I. (2002). Estrogen action via the G protein-coupled receptor, GPR30: stimulation of adenylyl cyclase and cAMP-mediated attenuation of the epidermal growth factor receptor-to-MAPK signaling axis. Mol. Endocrinol. 16 (1), 70–84. 10.1210/mend.16.1.0758 11773440

[B38] Franik Sle Q.-K. Kremer J. A. M. Kiesel L. Farquhar C. (2022). Aromatase inhibitors (letrozole) for ovulation induction in infertile women with polycystic ovary syndrome. Cochrane Database Syst. Rev. 2022 (9). 10.1002/14651858.cd010287.pub4 PMC9514207 36165742

[B39] Franks S. Hardy K. (2006). Development of polycystic ovary syndrome: involvement of genetic and environmental factors. Int. J. Androl. 29 (1), 86–90. discussion.278-85. 10.1111/j.1365-2605.2005.00623.x 16390494

[B40] Furuyama W. Enomoto M. Mossaad E. Kawai S. Mikoshiba K. Kawazu S. i. (2014). An interplay between 2 signaling pathways: melatonin-cAMP and IP3-Ca2+ signaling pathways control intraerythrocytic development of the malaria parasite Plasmodium falciparum. Biochem. biophysical Res. Commun. 446 (1), 125–131. 10.1016/j.bbrc.2014.02.070 24607908

[B41] Geissmann F. Jung S. Sieweke M. H. Merad M. Ley K. (2010). Development of monocytes, macrophages, and dendritic cells. Science 327 (5966), 656–661. 10.1126/science.1178331 20133564 PMC2887389

[B42] Gibson D. A. Saunders P. T. (2012). Estrogen dependent signaling in reproductive tissues - a role for estrogen receptors and estrogen related receptors. Mol. Cell. Endocrinol. 348 (2), 361–372. 10.1016/j.mce.2011.09.026 21964318

[B43] Giudice L. C. (2006). Endometrium in PCOS: implantation and predisposition to endocrine CA. Best Pract. Res. Clin. Endocrinol. metabolism 20 (2), 235–244. 10.1016/j.beem.2006.03.005 16772154

[B44] Gregory C. W. Wilson E. M. Apparao K. B. Lininger R. A. Meyer W. R. Kowalik A. (2002). Steroid receptor coactivator expression throughout the menstrual cycle in normal and abnormal endometrium. J. Clin. Endocrinol. metabolism 87 (6), 2960–2966. 10.1210/jcem.87.6.8572 12050280

[B45] Gu B. H. Park J. M. Baek K. H. (2010). Genetic variations of follicle stimulating hormone receptor are associated with polycystic ovary syndrome. Int. J. Mol. Med. 26 (1), 107–112. 10.3892/ijmm_00000441 20514429

[B46] Guang H. J. Li F. Shi J. (2018). Letrozole for patients with polycystic ovary syndrome: a retrospective study. Medicine 97 (44), e13038. 10.1097/MD.0000000000013038 30383669 PMC6221580

[B47] Hamza M. S. Ramadan E. Salama S. A. (2021). Glucose and fatty acid metabolism involved in the protective effect of metformin against ulipristal-induced endometrial changes in rats. Sci. Rep. 11 (1), 8863. 10.1038/s41598-021-88346-w 33893356 PMC8065147

[B48] Hayes M. G. Urbanek M. Ehrmann D. A. Armstrong L. L. Lee J. Y. Sisk R. (2015). Genome-wide association of polycystic ovary syndrome implicates alterations in gonadotropin secretion in European ancestry populations. Nat. Commun. 6, 7502. 10.1038/ncomms8502 26284813 PMC4557132

[B49] Heldring N. Pike A. Andersson S. Matthews J. Cheng G. Hartman J. (2007). Estrogen receptors: how do they signal and what are their targets. Physiol. Rev. 87 (3), 905–931. 10.1152/physrev.00026.2006 17615392

[B50] He S. Mao X. Lei H. Dong B. Guo D. Zheng B. (2020). Peripheral blood inflammatory-immune cells as a predictor of infertility in women with polycystic ovary syndrome. J. Inflamm. Res. 13, 441–450. 10.2147/JIR.S260770 32884325 PMC7443446

[B51] Hewitt S. C. Korach K. S. (2002). Estrogen receptors: structure, mechanisms and function. Rev. Endocr. metabolic Disord. 3 (3), 193–200. 10.1023/a:1020068224909 12215714

[B52] Hilborn E. Stal O. Jansson A. (2017). Estrogen and androgen-converting enzymes 17β-hydroxysteroid dehydrogenase and their involvement in cancer: with a special focus on 17β-hydroxysteroid dehydrogenase type 1, 2, and breast cancer. Oncotarget 8 (18), 30552–30562. 10.18632/oncotarget.15547 28430630 PMC5444764

[B53] Holte J. Bergh T. Berne C. Wide L. Lithell H. (1995). Restored insulin sensitivity but persistently increased early insulin secretion after weight loss in obese women with polycystic ovary syndrome. J. Clin. Endocrinol. metabolism 80 (9), 2586–2593. 10.1210/jcem.80.9.7673399 7673399

[B54] Ibanez L. Ong K. K. Mongan N. Jääskeläinen J. Marcos M. V. Hughes I. A. (2003). Androgen receptor gene CAG repeat polymorphism in the development of ovarian hyperandrogenism. J. Clin. Endocrinol. metabolism 88 (7), 3333–3338. 10.1210/jc.2002-021791 12843184

[B55] Jacquot Y. Spaggiari D. Schappler J. Lesniewska E. Rudaz S. Leclercq G. (2017). ERE-dependent transcription and cell proliferation: independency of these two processes mediated by the introduction of a sulfone function into the weak estrogen estrothiazine. Eur. J. Pharm. Sci. official J. Eur. Fed. Pharm. Sci. 109, 169–181. 10.1016/j.ejps.2017.07.026 28754571

[B56] Jayasena C. N. Franks S. (2014). The management of patients with polycystic ovary syndrome. Nat. Rev. Endocrinol. 10 (10), 624–636. 10.1038/nrendo.2014.102 25022814

[B57] Kahsar-Miller M. D. Nixon C. Boots L. R. Go R. C. Azziz R. (2001). Prevalence of polycystic ovary syndrome (PCOS) in first-degree relatives of patients with PCOS. Fertil. Steril. 75 (1), 53–58. 10.1016/s0015-0282(00)01662-9 11163816

[B58] Kahsar-Miller M. Azziz R. (1998). The development of the polycystic ovary syndrome: family history as a risk factor. Trends Endocrinol. metabolism TEM 9 (2), 55–58. 10.1016/s1043-2760(98)00021-6 18406241

[B59] Kato S. (2001). Estrogen receptor-mediated cross-talk with growth factor signaling pathways. Breast cancer 8 (1), 3–9. 10.1007/BF02967472 11180760

[B60] Kiddy D. S. Hamilton-Fairley D. Bush A. Short F. Anyaoku V. Reed M. J. (1992). Improvement in endocrine and ovarian function during dietary treatment of obese women with polycystic ovary syndrome. Clin. Endocrinol. 36 (1), 105–111. 10.1111/j.1365-2265.1992.tb02909.x 1559293

[B61] Knowlton A. A. Lee A. R. (2012). Estrogen and the cardiovascular system. Pharmacol. Ther. 135 (1), 54–70. 10.1016/j.pharmthera.2012.03.007 22484805 PMC5688223

[B62] Kosova G. Urbanek M. (2013). Genetics of the polycystic ovary syndrome. Mol. Cell. Endocrinol. 373 (1-2), 29–38. 10.1016/j.mce.2012.10.009 23079471 PMC3609918

[B63] Krentz A. J. Von Muhlen D. Barrett-Connor E. (2007). Searching for polycystic ovary syndrome in postmenopausal women: evidence of a dose-effect association with prevalent cardiovascular disease. Menopause 14 (2), 284–292. 10.1097/GME.0b013e31802cc7ab 17245231 PMC2642654

[B64] Kuiper G. G. Enmark E. Pelto-Huikko M. Nilsson S. Gustafsson J. A. (1996). Cloning of a novel receptor expressed in rat prostate and ovary. Proc. Natl. Acad. Sci. U. S. A. 93 (12), 5925–5930. 10.1073/pnas.93.12.5925 8650195 PMC39164

[B65] Kumar R. Balhuizen A. Amisten S. Lundquist I. Salehi A. (2011). Insulinotropic and antidiabetic effects of 17β-estradiol and the GPR30 agonist G-1 on human pancreatic islets. Endocrinology 152 (7), 2568–2579. 10.1210/en.2010-1361 21521748

[B66] Kurabayashi T. Suzuki M. Fujita K. Murakawa H. Hasegawa I. Tanaka K. (2006). Prognostic factors for ovulatory response with clomiphene citrate in polycystic ovary syndrome. Eur. J. obstetrics, Gynecol. reproductive Biol. 126 (2), 201–205. 10.1016/j.ejogrb.2005.11.005 16337728

[B67] Lau K. M. Laspina M. Long J. Ho S. M. (2000). Expression of estrogen receptor (ER)-alpha and ER-beta in normal and malignant prostatic epithelial cells: regulation by methylation and involvement in growth regulation. Cancer Res. 60 (12), 3175–3182. 10866308

[B68] Lau K. M. Leav I. Ho S. M. (1998). Rat estrogen receptor-alpha and -beta, and progesterone receptor mRNA expression in various prostatic lobes and microdissected normal and dysplastic epithelial tissues of the Noble rats. Endocrinology 139 (1), 424–427. 10.1210/endo.139.1.5809 9421443

[B69] Legrain S. Massien C. Lahlou N. Roger M. Debuire B. Diquet B. (2000). Dehydroepiandrosterone replacement administration: pharmacokinetic and pharmacodynamic studies in healthy elderly subjects. J. Clin. Endocrinol. metabolism 85 (9), 3208–3217. 10.1210/jcem.85.9.6805 10999810

[B70] Legro R. S. Brzyski R. G. Diamond M. P. Coutifaris C. Schlaff W. D. Casson P. (2014). Letrozole versus clomiphene for infertility in the polycystic ovary syndrome. N. Engl. J. Med. 371 (2), 119–129. 10.1056/NEJMoa1313517 25006718 PMC4175743

[B71] Legro R. S. Driscoll D. Strauss J. F. Fox J. Dunaif A. (1998). Evidence for a genetic basis for hyperandrogenemia in polycystic ovary syndrome. Proc. Natl. Acad. Sci. U. S. A. 95 (25), 14956–14960. 10.1073/pnas.95.25.14956 9843997 PMC24557

[B72] Li Y. R. Ren C. E. Zhang Q. Li J. C. Chian R. C. (2013). Expression of G protein estrogen receptor (GPER) on membrane of mouse oocytes during maturation. J. assisted reproduction Genet. 30 (2), 227–232. 10.1007/s10815-013-9942-z PMC3585672 23420106

[B73] Lindquist R. L. Shakhar G. Dudziak D. Wardemann H. Eisenreich T. Dustin M. L. (2004). Visualizing dendritic cell networks *in vivo* . Nat. Immunol. 5 (12), 1243–1250. 10.1038/ni1139 15543150

[B74] Liu Y. Yang K. Sun X. Fang P. Shi H. Xu J. (2015). MiR-138 suppresses airway smooth muscle cell proliferation through the PI3K/AKT signaling pathway by targeting PDK1. Exp. lung Res. 41 (7), 363–369. 10.3109/01902148.2015.1041581 26151666

[B75] Li Y. Birnbaumer L. Teng C. T. (2010). Regulation of ERRalpha gene expression by estrogen receptor agonists and antagonists in SKBR3 breast cancer cells: differential molecular mechanisms mediated by g protein-coupled receptor GPR30/GPER-1. Mol. Endocrinol. 24 (5), 969–980. 10.1210/me.2009-0148 20211987 PMC2870941

[B76] Lizneva D. Suturina L. Walker W. Brakta S. Gavrilova-Jordan L. Azziz R. (2016). Criteria, prevalence, and phenotypes of polycystic ovary syndrome. Fertil. Steril. 106 (1), 6–15. 10.1016/j.fertnstert.2016.05.003 27233760

[B77] Luan Y.-Y. Zhang L. Peng Y.-Q. Li Y. Y. Liu R. X. Yin C. H. (2022). Immune regulation in polycystic ovary syndrome. Clin. Chim. Acta 531, 265–272. 10.1016/j.cca.2022.04.234 35447143

[B78] Luu-The V. (2013). Assessment of steroidogenesis and steroidogenic enzyme functions. J. steroid Biochem. Mol. Biol. 137, 176–182. 10.1016/j.jsbmb.2013.05.017 23770321

[B79] Maneix L. Antonson P. Humire P. Rochel-Maia S. Castañeda J. Omoto Y. (2015). Estrogen receptor β exon 3-deleted mouse: the importance of non-ERE pathways in ERβ signaling. Proc. Natl. Acad. Sci. U. S. A. 112 (16), 5135–5140. 10.1073/pnas.1504944112 25848008 PMC4413313

[B80] March W. A. Moore V. M. Willson K. J. Phillips D. I. W. Norman R. J. Davies M. J. (2010). The prevalence of polycystic ovary syndrome in a community sample assessed under contrasting diagnostic criteria. Hum. Reprod. 25 (2), 544–551. 10.1093/humrep/dep399 19910321

[B81] Matsuzaki S. Fukaya T. Suzuki T. Murakami T. Sasano H. Yajima A. (1999). Oestrogen receptor alpha and beta mRNA expression in human endometrium throughout the menstrual cycle. Mol. Hum. Reprod. 5 (6), 559–564. 10.1093/molehr/5.6.559 10341004

[B82] Matthews J. Gustafsson J. A. (2003). Estrogen signaling: a subtle balance between ER alpha and ER beta. Mol. Interv. 3 (5), 281–292. 10.1124/mi.3.5.281 14993442

[B83] Mianowska B. Pietrzak I. Perenc M. Baranowska-Jaźwiecka A. Fendler W. Bodalski J. (2020). Sex hormones and insulin sensitivity in adolescent girls with type 1 diabetes. Diabetes Metab. 46 (1), 75–77. 10.1016/j.diabet.2018.07.004 30093281

[B84] Misso M. L. Wong J. L. Teede H. J. Hart R. Rombauts L. Melder A. M. (2012). Aromatase inhibitors for PCOS: a systematic review and meta-analysis. Hum. Reprod. update 18 (3), 301–312. 10.1093/humupd/dms003 22431566

[B85] Mitwally M. F. Casper R. F. (2001). Use of an aromatase inhibitor for induction of ovulation in patients with an inadequate response to clomiphene citrate. Fertil. Steril. 75 (2), 305–309. 10.1016/s0015-0282(00)01705-2 11172831

[B86] Moore A. M. Campbell R. E. (2017). Polycystic ovary syndrome: understanding the role of the brain. Front. Neuroendocrinol. 46, 1–14. 10.1016/j.yfrne.2017.05.002 28551304

[B87] Moravek M. B. Yin P. Ono M. Coon J. S. Dyson M. T. Navarro A. (2015). Ovarian steroids, stem cells and uterine leiomyoma: therapeutic implications. Hum. Reprod. update 21 (1), 1–12. 10.1093/humupd/dmu048 25205766 PMC4255606

[B88] Morley L. C. Tang T. Yasmin E. Norman R. J. Balen A. H. (2017). Insulin-sensitising drugs (metformin, rosiglitazone, pioglitazone, D-chiro-inositol) for women with polycystic ovary syndrome, oligo amenorrhoea and subfertility. Cochrane database Syst. Rev. 11, CD003053. CD003053. 10.1002/14651858.CD003053.pub6 29183107 PMC6486196

[B89] Mykhalchenko K. Lizneva D. Trofimova T. Walker W. Suturina L. Diamond M. P. (2017). Genetics of polycystic ovary syndrome. Expert Rev. Mol. diagnostics 17 (7), 723–733. 10.1080/14737159.2017.1340833 28602111

[B90] Naderpoor N. Shorakae S. De Courten B. Misso M. L. Moran L. J. Teede H. J. (2015). Metformin and lifestyle modification in polycystic ovary syndrome: systematic review and meta-analysis. Hum. Reprod. update 21 (5), 560–574. 10.1093/humupd/dmv025 26060208

[B91] Nilsson S. Gustafsson J. A. (2011). Estrogen receptors: therapies targeted to receptor subtypes. Clin. Pharmacol. Ther. 89 (1), 44–55. 10.1038/clpt.2010.226 21124311

[B92] Niro S. Pereira E. PéLISSIER M.-A. Robert M. Olivier H. (2012). The DHEA metabolite 7β-hydroxy-epiandrosterone exerts anti-estrogenic effects on breast cancer cell lines. Steroids 77 (5), 542–551. 10.1016/j.steroids.2012.01.019 22342541

[B93] Norman R. J. Masters L. Milner C. R. Wang J. X. Davies M. J. (2001). Relative risk of conversion from normoglycaemia to impaired glucose tolerance or non-insulin dependent diabetes mellitus in polycystic ovarian syndrome. Hum. Reprod. 16 (9), 1995–1998. 10.1093/humrep/16.9.1995 11527911

[B94] Paeeh K. Webb P. Kuiper G. G. Nilsson S. Gustafsssn J. Kushner P. J. (1997). Differential ligand activation of estrogen receptors ERalpha and ERbeta at AP1 sites. Science 277 (5331), 1508–1510. 10.1126/science.277.5331.1508 9278514

[B95] Palomba Spiltonen T. T. Giudice L. C. (2021). Endometrial function in women with polycystic ovary syndrome: a comprehensive review. Hum. Reprod. Update 27 (3), 584–618. 10.1093/humupd/dmaa051 33302299

[B96] Pang Y. Thomas P. (2010). Role of G protein-coupled estrogen receptor 1, GPER, in inhibition of oocyte maturation by endogenous estrogens in zebrafish. Dev. Biol. 342 (2), 194–206. 10.1016/j.ydbio.2010.03.027 20382141 PMC2874603

[B97] Paramanik V. Krishnan H. Kumar Thakur M. (2018). Estrogen receptor α- and β-interacting proteins contain consensus secondary structures: an insilico study. Ann. Neurosci. 25 (1), 1–10. 10.1159/000481809 29887678 PMC5981639

[B98] Paterni I. Granchi C. Katzenellenbogen J. A. Minutolo F. (2014). Estrogen receptors alpha (ERα) and beta (ERβ): subtype-selective ligands and clinical potential. Steroids 90, 13–29. 10.1016/j.steroids.2014.06.012 24971815 PMC4192010

[B99] Prossnitz E. R. Barton M. (2011). The G-protein-coupled estrogen receptor GPER in health and disease. Nat. Rev. Endocrinol. 7 (12), 715–726. 10.1038/nrendo.2011.122 21844907 PMC3474542

[B100] Prossnitz E. R. Barton M. (2014). Estrogen biology: new insights into GPER function and clinical opportunities. Mol. Cell. Endocrinol. 389 (1-2), 71–83. 10.1016/j.mce.2014.02.002 24530924 PMC4040308

[B101] Prossnitz E. R. Barton M. (2023). The G protein-coupled oestrogen receptor GPER in health and disease: an update. Nat. Rev. Endocrinol. 19 (7), 407–424. 10.1038/s41574-023-00822-7 37193881 PMC10187525

[B102] Pusalkar M. Meherji P. Gokral J. Chinnaraj S. Maitra A. (2009). CYP11A1 and CYP17 promoter polymorphisms associate with hyperandrogenemia in polycystic ovary syndrome. Fertil. Steril. 92 (2), 653–659. 10.1016/j.fertnstert.2008.07.016 18725155

[B103] Qiu Jronnekleiv O. K. Kelly M. J. (2008). Modulation of hypothalamic neuronal activity through a novel G-protein-coupled estrogen membrane receptor. Steroids 73 (9-10), 985–991. 10.1016/j.steroids.2007.11.008 18342349 PMC5466077

[B104] Quezada S. Avellaira C. Johnson M. C. Gabler F. Fuentes A. Vega M. (2006). Evaluation of steroid receptors, coregulators, and molecules associated with uterine receptivity in secretory endometria from untreated women with polycystic ovary syndrome. Fertil. Steril. 85 (4), 1017–1026. 10.1016/j.fertnstert.2005.09.053 16580389

[B105] Ramos Cirilo P. D. Rosa F. E. Moreira Ferraz M. F. Rainho C. A. Pontes A. Rogatto S. R. (2012). Genetic polymorphisms associated with steroids metabolism and insulin action in polycystic ovary syndrome. Gynecol. Endocrinol. official J. Int. Soc. Gynecol. Endocrinol. 28 (3), 190–194. 10.3109/09513590.2011.593661 21824047

[B106] Rasmussen M. Ekstrand B. Zamaratskaia G. (2013). Regulation of 3β-hydroxysteroid dehydrogenase/Δ⁵-Δ⁴ isomerase: a review. Int. J. Mol. Sci. 14 (9), 17926–17942. 10.3390/ijms140917926 24002028 PMC3794760

[B107] Revankar C. M. Cimino D. F. Sklar L. A. Arterburn J. B. Prossnitz E. R. (2005). A transmembrane intracellular estrogen receptor mediates rapid cell signaling. Science 307 (5715), 1625–1630. 10.1126/science.1106943 15705806

[B108] Reyes-Munoz E. Sathyapalan T. Rossetti P. Shah M. Long M. Buscema M. (2018). Polycystic ovary syndrome: implication for drug metabolism on assisted reproductive techniques-A literature review. Adv. Ther. 35 (11), 1805–1815. 10.1007/s12325-018-0810-1 30311070 PMC6224003

[B109] Roque M. Tostes A. C. Valle M. Sampaio M. Geber S. (2015). Letrozole versus clomiphene citrate in polycystic ovary syndrome: systematic review and meta-analysis. Gynecol. Endocrinol. official J. Int. Soc. Gynecol. Endocrinol. 31 (12), 917–921. 10.3109/09513590.2015.1096337 26479460

[B110] Rosenfield R. L. Ehrmann D. A. (2016). The pathogenesis of polycystic ovary syndrome (PCOS): the hypothesis of PCOS as functional ovarian hyperandrogenism revisited. Endocr. Rev. 37 (5), 467–520. 10.1210/er.2015-1104 27459230 PMC5045492

[B111] Rotterdam E. (2004). Revised 2003 consensus on diagnostic criteria and long-term health risks related to polycystic ovary syndrome (PCOS). Hum. Reprod. 19, 41–47. 10.1093/humrep/deh098 14688154

[B112] Rotterdam ESHRE/ASRM-Sponsored PCOS consensus workshop group (2004). Revised 2003 consensus on diagnostic criteria and long-term health risks related to polycystic ovary syndrome. Fertil. Steril. 81 (1), 19–25. 10.1016/j.fertnstert.2003.10.004 14711538

[B113] Ryu Y. Kim S. W. Kim Y. Y. Ku S. Y. (2019). Animal models for human polycystic ovary syndrome (PCOS) focused on the use of indirect hormonal perturbations: a review of the literature. Int. J. Mol. Sci. 20 (11), 2720. 10.3390/ijms20112720 31163591 PMC6600358

[B114] Shamsi M. Ghazavi Asaeedifar A. M. Mosayebi G. Pour S. K. Ganji A. (2022). The immune system’s role in PCOS. Mol. Biol. Rep. 49 (11), 10689–10702. 10.1007/s11033-022-07695-5 35752698

[B115] Sharma G. Prossnitz E. R. (2011). Mechanisms of estradiol-induced insulin secretion by the G protein-coupled estrogen receptor GPR30/GPER in pancreatic beta-cells. Endocrinology 152 (8), 3030–3039. 10.1210/en.2011-0091 21673097 PMC3138237

[B116] Shi Y. Zhao H. Shi Y. Cao Y. Yang D. Li Z. (2012). Genome-wide association study identifies eight new risk loci for polycystic ovary syndrome. Nat. Genet. 44 (9), 1020–1025. 10.1038/ng.2384 22885925

[B117] Shrivastava S. Conigliaro R. L. (2023). Polycystic ovarian syndrome. Med. Clin. North Am. 107 (2), 227–234. 10.1016/j.mcna.2022.10.004 36759093

[B118] Silva F. S. Soter M. O. Sales M. F. Candido A. L. Reis F. M. Silva I. F. O. (2015). Estrogen receptor αlpha gene (ESR1) PvuII and XbaI polymorphisms are associated to metabolic and proinflammatory factors in polycystic ovary syndrome. Gene 560 (1), 44–49. 10.1016/j.gene.2015.01.037 25617525

[B119] Stein I. F. Leventhai M. L. (1935). Amenorrhea associated with bilateral polycystic ovaries. Am. J. Obstet. Gynecol. 29, 181–191. 10.1016/s0002-9378(15)30642-6

[B120] Stocco C. (2008). Aromatase expression in the ovary: hormonal and molecular regulation. Steroids 73 (5), 473–487. 10.1016/j.steroids.2008.01.017 18321551 PMC2365984

[B121] Su N. J. Ma J. Feng D. F. Zhou S. Li Z. T. Zhou W. P. (2018). The peripheral blood transcriptome identifies dysregulation of inflammatory response genes in polycystic ovary syndrome. Gynecol. Endocrinol. 34 (7), 584–588. 10.1080/09513590.2017.1418851 29262729

[B122] Tagliaferri V. Romualdi D. Scarinci E. Cicco S. D. Florio C. D. Immediata V. (2018). Melatonin treatment may Be able to restore menstrual cyclicity in women with PCOS: a pilot study. Reprod. Sci. 25 (2), 269–275. 10.1177/1933719117711262 28558523

[B123] Talpur H. S. Worku T. Rehman Z. U. Dad R. Bhattarai D. Bano I. (2017). Knockdown of melatonin receptor 1 and induction of follicle-stimulating hormone on the regulation of mouse granulosa cell function. Reprod. Biol. 17 (4), 380–388. 10.1016/j.repbio.2017.10.005 29097083

[B124] Tang Z. R. Zhang R. Lian Z. X. Deng S. L. Yu K. (2019). Estrogen-receptor expression and function in female reproductive disease. Cells 8 (10), 1123. 10.3390/cells8101123 31546660 PMC6830311

[B125] Taniguchi Fcouse J. F. Rodriguez K. F. Emmen J. M. A. Poirier D. Korach K. S. (2007). Estrogen receptor-alpha mediates an intraovarian negative feedback loop on thecal cell steroidogenesis via modulation of Cyp17a1 (cytochrome P450, steroid 17alpha-hydroxylase/17,20 lyase) expression. FASEB J. official Publ. Fed. Am. Soc. Exp. Biol. 21 (2), 586–595. 10.1096/fj.06-6681com PMC1896370 17158782

[B126] Teede H. J. Tay C. T. Laven J. Dokras A. Moran L. J. Piltonen T. T. (2023). Recommendations from the 2023 international evidence-based guideline for the assessment and management of polycystic ovary syndrome. Hum. Reprod. 38 (9), 1655–1679. 10.1093/humrep/dead156 37580037 PMC10477934

[B127] Tremblay A. Giguere V. (2001). Contribution of steroid receptor coactivator-1 and CREB binding protein in ligand-independent activity of estrogen receptor beta. J. steroid Biochem. Mol. Biol. 77 (1), 19–27. 10.1016/s0960-0760(01)00031-0 11358671

[B128] Tskitishvili E. Pequeux C. Munaut C. Viellevoye R. Nisolle M. Noël A. (2017). Estrogen receptors and estetrol-dependent neuroprotective actions: a pilot study. J. Endocrinol. 232 (1), 85–95. 10.1530/JOE-16-0434 27799463 PMC5118942

[B129] Ueda S. Tsuda H. Sato K. Takeuchi H. Shigekawa T. Matsubara O. (2006). Alternative tyrosine phosphorylation of signaling kinases according to hormone receptor status in breast cancer overexpressing the insulin-like growth factor receptor type 1. Cancer Sci. 97 (7), 597–604. 10.1111/j.1349-7006.2006.00228.x 16827799 PMC11158992

[B130] Unsal T. Konac E. Yesilkaya E. Yilmaz A. Bideci A. Ilke Onen H. (2009). Genetic polymorphisms of FSHR, CYP17, CYP1A1, CAPN10, INSR, SERPINE1 genes in adolescent girls with polycystic ovary syndrome. J. assisted reproduction Genet. 26 (4), 205–216. 10.1007/s10815-009-9308-8 PMC2682189 19387820

[B131] Vihko P. Isomaa V. Ghosh D. (2001). Structure and function of 17beta-hydroxysteroid dehydrogenase type 1 and type 2. Mol. Cell. Endocrinol. 171 (1-2), 71–76. 10.1016/s0303-7207(00)00389-0 11165013

[B132] Visniauskas B. Kilanowski-Doroh I. Ogola B. O. Mcnally A. B. Horton A. C. Sugi A. I. (2023). Estrogen-mediated mechanisms in hypertension and other cardiovascular diseases. J. Hum. Hypertens. 37 (8), 609–618. 10.1038/s41371-022-00771-0 36319856 PMC10919324

[B133] Visser M. Coelingh Bennink H. J. (2008). *In vitro* effects of estetrol on receptor binding, drug targets and human liver cell metabolism. Climacteric J. Int. Menopause Soc. 1, 64–68. 10.1080/13697130802050340 18464025

[B134] Wang C. Roy S. K. (2008). G protein-coupled receptor 30 expression is required for estrogen stimulation of primordial follicle formation in the hamster ovary. Endocrinology 149 (9), 4452–4461. 10.1210/en.2008-0441 18499747 PMC2553386

[B135] Warmerdam E. G. Visser M. Coelingh Bennink H. J. Groen M. (2008). A new route of synthesis of estetrol. Climacteric J. Int. Menopause Soc. 1, 59–63. 10.1080/13697130802054078 18464024

[B136] Wu W. LoVerde P. T. (2019). Nuclear hormone receptors in parasitic Platyhelminths. Mol. Biochem. Parasitol. 233, 111218. 10.1016/j.molbiopara.2019.111218 31470045 PMC6783382

[B137] Yildiz B. O. Bolour S. Woods K. Moore A. Azziz R. (2010). Visually scoring hirsutism. Hum. Reprod. update 16 (1), 51–64. 10.1093/humupd/dmp024 19567450 PMC2792145

[B138] Yildiz B. O. Bozdag G. Yapici Z. Esinler I. Yarali H. (2012). Prevalence, phenotype and cardiometabolic risk of polycystic ovary syndrome under different diagnostic criteria. Hum. Reprod. 27 (10), 3067–3073. 10.1093/humrep/des232 22777527

[B139] Yu K. Huang Z. Y. Xu X. L. Li J. Fu X. W. Deng S. L. (2022). Estrogen receptor function: impact on the human endometrium. Front. Endocrinol. Lausanne 13, 827724. 10.3389/fendo.2022.827724 35295981 PMC8920307

[B140] Yu K. Wang R. X. Li M. H. Sun T. C. Zhou Y. W. Li Y. Y. (2019). Melatonin reduces androgen production and upregulates heme oxygenase-1 expression in granulosa cells from PCOS patients with hypoestrogenia and hyperandrogenia. Oxid. Med. Cell Longev. 2019, 8218650. accepted manuscript.2019:8218650. 10.1155/2019/8218650 31772710 PMC6854986

[B141] Zawadzki J. Dunaif A. (1992). “Diagnostic criteria for polycystic syndrome: towards a rational approach,” in Polycystic ovary syndrome Editors Dunaif A. Givens J. R. Haseltine F. P. (Boston: Blackwell Scientific), 337–384.

[B142] Zhang T. Tian F. Huo R. Tang A. Zeng Y. Duan Y. G. (2017). Detection of dendritic cells and related cytokines in follicular fluid of patients with polycystic ovary syndrome. Am. J. Reproductive Immunol. 78 (3). 10.1111/aji.12717 28585716

[B143] Zhao H. L. V. Y. Li L. Chen Z. J. (2016). Genetic studies on polycystic ovary syndrome. Best Pract. Res. Clin. obstetrics Gynaecol. 37, 56–65. 10.1016/j.bpobgyn.2016.04.002 27264388

[B144] Zhou Z. Moore T. M. Drew B. G. Ribas V. Wanagat J. Civelek M. (2020). Estrogen receptor α controls metabolism in white and brown adipocytes by regulating Polg1 and mitochondrial remodeling. Sci. Transl. Med. 12 (555), eaax8096. 10.1126/scitranslmed.aax8096 32759275 PMC8212422

